# BioNetStat: A Tool for Biological Networks Differential Analysis

**DOI:** 10.3389/fgene.2019.00594

**Published:** 2019-06-21

**Authors:** Vinícius Carvalho Jardim, Suzana de Siqueira Santos, Andre Fujita, Marcos Silveira Buckeridge

**Affiliations:** ^1^Department of Computer Science, Institute of Mathematics and Statistics, University of São Paulo, São Paulo, Brazil; ^2^Department of Botany, Institute of Biosciences, University of São Paulo, São Paulo, Brazil

**Keywords:** differential network analysis, coexpression network, correlation network, systems biology, systems biology tool, differential coexpression, differential correlation

## Abstract

The study of interactions among biological components can be carried out by using methods grounded on network theory. Most of these methods focus on the comparison of two biological networks (e.g., control vs. disease). However, biological systems often present more than two biological states (e.g., tumor grades). To compare two or more networks simultaneously, we developed BioNetStat, a Bioconductor package with a user-friendly graphical interface. BioNetStat compares correlation networks based on the probability distribution of a feature of the graph (e.g., centrality measures). The analysis of the structural alterations on the network reveals significant modifications in the system. For example, the analysis of centrality measures provides information about how the relevance of the nodes changes among the biological states. We evaluated the performance of BioNetStat in both, toy models and two case studies. The latter related to gene expression of tumor cells and plant metabolism. Results based on simulated scenarios suggest that the statistical power of BioNetStat is less sensitive to the increase of the number of networks than Gene Set Coexpression Analysis (GSCA). Also, besides being able to identify nodes with modified centralities, BioNetStat identified altered networks associated with signaling pathways that were not identified by other methods.

## 1. Introduction

In the last two decades, the high-dimensional data production, such as metabolomics, proteomics, transcriptomics, and genomics, increased considerably (Zhu et al., 2008; McKenzie et al., 2016). It brings out the high complexity of the biological systems, posing the challenge to understand how they work. In science, it is fundamental to compare the many states assumed by a system, such as sick against healthy patients or developmental stages of a living being. A range of strategies can be applied for comparing different states depending on the study hypothesis, such as the *t-*test (to compare two means), the analysis of variance—ANOVA (to compare two or more means) (de Souza et al., 2008; Wu et al., 2016) or Gene Set Enrichment Analysis (GSEA), to test whether a gene set is differentially expressed between two conditions (Subramanian et al., 2005). However, none of these methods takes into account the relationship among several biological components at the same time. In this sense, methods based on networks represent the association between each pair of components and may help to understand the role each variable plays in the system (Barabási and Oltvai, 2004).

Biological systems can be assessed by correlation networks, in which the nodes represent the elements (variables) and edges represent the statistical relations among its elements. Some approaches have been proposed to qualitatively analyze the correlation networks by performing a visual inspection of their structure (Caldana et al., 2011; Weston et al., 2011), while others are based on formal strategies to search for differences between biological networks (Sun et al., 2013; Li et al., 2016; Zhang and Yin, 2016). However, these studies do not apply statistical tests or formal control of false positives.

Over the last years, several tools have been developed to statistically test whether correlation networks are different across conditions. Examples include DCGL (Liu et al., 2010), EBcoexpress (Dawson et al., 2012), DiffCorr (Fukushima, 2013), and CoDiNA (Gysi et al., 2018), which evaluate whether the correlations between pairs of nodes are different among biological states. DiffCoEx (Tesson et al., 2010) coXpress (Watson, 2006) searches for cohesive subgroups of variables in one of the states and evaluates whether these groups change their correlation patterns among states. DINGO (Ha et al., 2015), DECODE (Lui et al., 2015), dCoxS (Cho et al., 2009), GSCA (Choi and Kendziorski, 2009), GSNCA (Rahmatallah et al., 2014), and CoGA (Santos et al., 2015) compare predefined sets of variables (Santos et al., 2015). Here we focus on the last group, in which the tests are performed for each predefined group of variables.

Although several biological studies compare more than two networks (Caldana et al., 2011; Weston et al., 2011; Hochberg et al., 2013; Zhang and Yin, 2016), to the best of our knowledge, there are only two tools that perform statistical tests to compare two or more networks simultaneously: DiffCoEx and GSCA. However, only GSCA performs tests for predefined groups of variables. GSCA builds correlations matrices and compares the biological condition networks by using Euclidean distance (Choi and Kendziorski, 2009). Pairwise comparison between the networks obtains the GSCA generalization for comparing more than two networks. However, this strategy, in general, gives an inadequate control of type I error (Fujita et al., 2017). Besides, since the network structure may vary over time and also across systems from the same biological class, searching for precisely similar structures between two graphs is not an effective strategy to compare the behavior of biological pathways (Santos et al., 2015).

In the context of functional brain network studies, a generalization of CoGA, named by GANOVA, has been proposed to compare more than two populations of graphs (Fujita et al., 2017). This tool is specific for datasets containing several graphs in each biological condition. GANOVA is not useful when only one network is available per condition, such as in the case of physiological or genes correlations networks. Here we combined the methods proposed by Santos et al. (2015) and Fujita et al. (2017) to compare two or more biological states, namely BioNetStat. BioNetStat is available at Bioconductor and includes a graphical user interface. We performed simulation experiments and applied the proposed method in two biological data sets.

## 2. Materials and Methods

We propose a method for comparing simultaneously two or more biological correlation networks. In the following subsections, we explain the construction of correlation networks (graphs), the structural graph analysis, and the statistical test performed by BioNetStat.

### 2.1. Construction of Correlation Networks

A correlation network is an undirected graph, where each node corresponds to a biological variable, and each edge connects a pair of nodes indicating the association between two variables. In our context, the edge corresponds to the statistical dependence between two variables. To measure and detect monotonic relations, BioNetStat includes the Pearson (1920), Spearman (1904), and Kendall (1938) correlation coefficients. Given a measure of statistical dependence, BioNetStat provides three scales of association degree: the absolute correlation coefficient, one minus the *p*-value of the dependence test, and one minus the *p*-value adjusted by the False Discovery Rate method (Benjamini and Hochberg, 1995). Each association degree is a real number varying from zero to one. The user can choose between unweighted (zero or one) and weighted network (values from zero to one). Zero means no monotonic association between variables, while one means a monotonic association between them. To construct a graph, the user can choose a threshold for edges insertion, based on some association measure (correlation or *p*-value of the independence test).

The proposed method is based on graph topological features. In the following sections, we describe how BioNetStat performs the comparisons based in the Probability Distribution of a Feature of the Graph (PDFG), in the vector of some network centrality, and in each node centrality measure.

### 2.2. Differential Network Analysis of Multiple Graphs Based on PDFG

A random graph *G* is a graph generated by a random process. In the last decades, several random graph models have been proposed for studying biological networks. For example, Barabasi and Albert (1999) proposed the scale-free model, in which a few nodes have many connections (hubs) and many nodes present a lower number of connections (Jeong et al., 2000). An example where to which the scale-free model suits well is in the representation of the protein-protein interactions networks, in which only a few essential proteins interact with many others and are central to metabolism, whereas many proteins display lower numbers of interactions because they participate in a few specific metabolic pathways.

Consider a set of nodes *V* = {*v*_1_, *v*_2_, …, *v*_*n*_*v*__} of the graph, *r* states *S*_1_, *S*_2_, …, *S*_*r*_, and *o*_*i*_ samples (number of observations) for each state *S*_*i*_, for *i* = 1, 2, …, *r*. We want to test whether the *r* graphs *G*_1_, *G*_2_, …, *G*_*r*_ (each one representing a state) were generated by the same random graph model. In case the PDFG are different, it would be assumed that the graphs were generated by different random graph models. As will be seen next, here we analyzed correlation networks in which the elements correspond to variables such as genes, proteins, metabolites, and phenotypic variables. Examples of states include different treatments or conditions. An alteration in the structure of the network, detected by a change in the PDFG, could mean that a healthy human cell may be turning into a tumor cell or the tumor tissue might be entering in a new degree of aggressiveness.

The *differential network analysis* consists of the following steps: (i) construction of a correlation network for each state, which are denoted by *G*_1_, *G*_2_, …, *G*_*r*_, (ii) computation of the statistic test, denoted by θ, which quantifies the differences among the networks, and (iii) a permutation test.

The PDFG is the probability density function of some topological feature *x* and has *n*_*v*_ elements *x*_1_, *x*_2_, …, *x*_*n*_*v*__. Examples of topological features are the set of eigenvalues of the adjacency matrix of the graph, or graph centrality measures. Let δ be the Dirac's delta and the brackets “〈〉” denote the expectation according to the probability law of a random graph. Formally, the PDFG (*g*) is defined as:

(1) $$\begin{array}{r} \rho_{g}\left(x\right)=\underset{n_{v}\to \infty }{\lim}\langle \frac{1}{n_{v}}\overset{n_{v}}{\underset{j=1}{\sum }}\delta \left(x-x_{j}/\sqrt{n_{v}}\right)\rangle \end{array}$$

In real systems, the PDFG is unknown. To estimate the PDFG, BioNetStat uses the Gaussian Kernel estimator implemented by the function *density* of the R base package. The user can choose between the Sturges' (Sturges, 1926) and the Silverman's (Silverman, 1986) criteria to define the Kernel bandwidth for the Gaussian Kernel estimator. In the analyses performed in this work, we used the Sturges' criterion.

#### 2.2.1. Computation of the Statistic Test

The *differential network analysis* is a comparison between two or more graphs based on their PDFG.

The θ statistic is calculated as follows:

1. For each graph *G*_*i*_ (*i* = 1, …, *r*), compute the PDFG denoted by ρ_*g*_*i*__.
2. Calculate the average PDFG as:
  (2) $$\begin{array}{r} \rho_{g_{M}}=\frac{\sum_{i=1}^{r}\rho_{g_{i}}}{r}. \end{array}$$
3. Calculate the Kullback-Leiber (KL) divergence between (*ρ*_*g*_*i*__) and *ρ*_*g*_*M*__ :
  (3) $$\begin{array}{r} D_{i}=KL\left(\rho_{g_{i}}|\rho_{g_{M}}\right) \end{array}$$
4. The statistic θ, which measures the difference among graphs, is the average distance:
  (4) $$\begin{array}{r} \theta =\frac{\sum_{i=1}^{r}D_{i}}{r}. \end{array}$$

The KL divergence measures the discrepancy between two probability distributions. For graphs, we can use the KL divergence to select the graph model that best describes the observed graph or to discriminate PDFGs (Takahashi et al., 2012). Formally, we define the KL divergence between graphs as follows. Let *g*_1_ and *g*_2_ be two random graphs with densities *ρ*_*g*_1__ and *ρ*_*g*_2__, respectively. If the support of *ρ*_*g*_2__ contains the support of *ρ*_*g*_1__, then the KL divergence between *ρ*_*g*_1__ and *ρ*_*g*_2__ is (Takahashi et al., 2012):

(5) $$\begin{array}{r} KL\left(\rho_{g_{1}}|\rho_{g_{2}}\right)=-\int_{-\infty }^{+\infty }\rho_{g_{1}}\left(x\right)\log\frac{\rho_{g_{1}}\left(x\right)}{\rho_{g_{2}}\left(x\right)}dx \end{array}$$

where 0 log 0 = 0 and *ρ*_*g*_2__ is called the reference measure. If the support of *ρ*_*g*_2__ does not contain the support of *ρ*_*g*_1__, then *KL*(*ρ*_*g*_1__|*ρ*_*g*_2__) = +∞. The KL divergence is non-negative, and it is zero if and only if *ρ*_*g*_1__ and *ρ*_*g*_2__ are equal. For many cases, *KL*(*ρ*_*g*_1__|*ρ*_*g*_2__) and *KL*(*ρ*_*g*_2__|*ρ*_*g*_1__) are different when *ρ*_*g*_1__ and *ρ*_*g*_2__ are not equal, i.e., KL is an asymmetric measure.

### 2.3. Differential Network Analysis of Multiple Graphs Based on Graph Centralities

As in section 2.2, consider a set of nodes *V* = {*v*_1_, *v*_2_, …, *v*_*n*_*v*__} and a set of edges *E* = {*e*_1_, *e*_2_, …, *e*_*n*_*e*__} of the graph, *r* states *S*_1_, *S*_2_, …, *S*_*r*_, and *o*_*i*_ samples (number of observations) of each state *S*_*i*_, for *i* = 1, 2, …, *r*. The aim is to test if the centrality values of *r* graphs *G*_1_, *G*_2_, …, *G*_*r*_, of each state, are the same among all graphs. BioNetStat considers five node centrality measures, namely degree, eigenvector, closeness, betweenness, and clustering coefficient, and one edge centrality (edge betweenness). The centrality measures quantify the importance of each node/edge according to its position in the network. The degree centrality counts the number of connections of a node (Barabási and Oltvai, 2004). In correlation networks, a node with high degree centrality is correlated with several other nodes/variables. This, such a node may be involved in numerous biological processes. The eigenvector centrality of a node is proportional to the centralities of its neighbors weighted by the strength of the connections (Bonacich, 1972). That is, a node is progressively more important as it connects with higher numbers of strongly connected neighbors nodes. The closeness and betweenness centralities are related to the shortest paths in the network (Rubinov and Sporns, 2010). The closeness centrality measures the average proximity of a node to all other nodes (Freeman, 1978). The betweenness centrality measures the importance of a node in the communication of the network. It counts how many shortest paths pass through the node (Freeman, 1978). The clustering coefficient quantifies how connected the neighbors of a node are (Watts and Strogatz, 1998). Finally, the edge betweenness centrality is similar to the betweenness centrality for nodes (Girvan and Newman, 2002). It quantifies how many shortest paths pass through an edge, measuring its importance in the communication of the network. The mathematical definitions of these six measures are shown in the Table S5.

Alterations in the centrality measures among networks means that the importance of the gene/protein/metabolite changed, i.e., its connectivity was altered regarding the main issues associated. Our tool, therefore, affords evaluation of data by assessing: (i) importance of a node in relation to the entire population of nodes in the network; (ii) proximity among nodes; (iii) importance of a node in the communication within the network, and (iv) the connectivity strength of the network as a whole.

The differential analysis consists of the same steps described in section 2.2.1. However, since in this case we are comparing the graphs centralities, the PDFG *ρ*_*g*_*i*__ is replaced by the vector of centrality measure and the *D*_*i*_ by the Euclidean distance between the vector of nodes/edges centralities of graph *G*_*i*_ and the vector containing the average centralities among the graphs (steps 2 and 3 of section 2.2.1).

### 2.4. Differential Node Analysis of Multiple Graphs Based on Node Centralities

Consider a set of nodes *V* = {*v*_1_, *v*_2_, …, *v*_*n*_*v*__} and a set of edges *E* = {*e*_1_, *e*_2_, …, *e*_*n*_*e*__} of the graph, *r* states *S*_1_, *S*_2_, …, *S*_*r*_, and *o*_*i*_ samples (number of observations) of each state *S*_*i*_, for *i* = 1, 2, …, *r*. The aim is to test if the importance (centrality value) of node *v*_*j*_, for *j* = 1, 2, …, *n*_*v*_, or for the edge *e*_*l*_, for *l* = 1, 2, …, *n*_*e*_, is the same among *r* graphs *G*_1_, *G*_2_, …, *G*_*r*_, of each state. In the same way that was done in section 2.3, here we considers the five node centrality measures (degree, eigenvector, closeness, betweenness, and clustering coefficient) and the edge centrality (edge betweenness).

The *differential node analysis* consists in similar steps as in section 2.2: (i) construction of a correlation network for each state, which are denoted by *G*_1_, *G*_2_, …, *G*_*r*_, (ii) computation of the statistic test, denoted by θ, which quantifies the differences among the node centralities of each network, and (iii) a permutation test.

#### 2.4.1. Computation of the Test Statistic for Node Comparison

The θ statistic is calculated as follows:

1. For each node *V*_*j*_ (*j* = 1, …, *n*_*v*_) or for each edge *E*_*l*_ (*l* = 1, …, *e*_*v*_) in graph *G*_*i*_ (*i* = 1, …, *r*), compute the node centrality denoted by $C_{i}^{j}$, or edge centrality, replacing *j* for *l*.
2. From the *r* centralities of each node/edge in each graph, we obtain an average node/edge centrality as:
  (6) $$\begin{array}{r} M^{j}=\frac{\sum_{i=1}^{r}C_{i}^{j}}{r}. \end{array}$$
3. Calculate the distance between the centrality of nodes/edges in each graph *G*_*i*_ $\left(C_{i}^{j}\right)$ and the average node/edge centrality (*M*^*j*^):
  (7) $$\begin{array}{r} D_{i}^{j}=|C_{i}^{j}-M^{j}|. \end{array}$$
4. The statistic θ, which measures the difference among centralities for each node/edge *j* of graphs, is the average distance:
  (8) $$\begin{array}{r} \theta =\frac{\sum_{i=1}^{r}D_{i}^{j}}{r}. \end{array}$$

### 2.5. Permutation Test

The hypotheses to be tested are defined as:

*H*_0_ : θ = 0 vs. *H*_1_ :θ > 0.

To construct the null hypothesis we perform a permutation test as follows:

1. Compute $\hat{\theta }$.
2. Construct *r* new graphs by resampling the observations without replacement.
3. Compute ${\hat{\theta }}^{*}$ by using the graphs constructed in step 2.
4. Repeat steps 2 and 3 until obtaining the desired number of permutation replications.
5. Test if $\hat{\theta }=0$ using the empirical distribution obtained in steps 2–4. Gather the information from the empirical distribution of ${\hat{\theta }}^{*}$ to obtain a *p*-value for $\hat{\theta }=0$, by analyzing the probability of obtaining values equal or greater than $\hat{\theta }$.

### 2.6. Description of the BioNetStat Package

BioNetStat is implemented in R http://cran.r-project.org/, provides a graphical interface, and is used to study correlation networks. It is based on the following packages: (i) CoGA to calculate the PDFG measures and the Kullback-Leibler divergence; (ii) shiny, shinyBS, yaml, whisker, and RJSONIO for browser interface; (iii) igraph to compute graph topological properties; (iv) Hmisc and psych for graph inference; and (v) ggplot2, pathview, pheatmap, and RColorBrewer for plotting.

BioNetStat receives two files as input. One is the *Biological samples file*, with the pre-processed data, containing the values of the variables (e.g. gene expression levels or metabolites concentration). This file must be a table, in which the columns indicate the variables and rows indicate the biological samples. At least one of these columns should indicate the label of rows (e.g. state to which each biological sample is related to). A second file, *variable set file*, contains the pre-defined set of variables (e.g., sets of biological variables belonging to the same metabolic pathways, sharing the same Gene Ontology terms). As an example of gene set collections, we suggest the use of Molecular Signature Database (MSigDB in http://www.broadinstitute.org/gsea/msigdb/index.jsp) (Subramanian et al., 2005), which is available for download.

For *differential network analysis*, presented in sections 2.2 and 2.3, BioNetStat returns a table containing the set name, the number of compared graphs, the size of each set, the statistics of the test, the permutation-based *p*-values, and the adjusted *p*-values by False Discovery Rate method (Benjamini and Hochberg, 1995) for multiple tests (*q*-values). An example of the output is shown in Supplementary Data Sheet 1. If the user performs the node differential analysis (section 2.4), the software returns a table containing the variable name, the statistics of the test, the permutation-based *p*-values, the *q*-values, and the node/edge centrality in each network, as shown in Table 1.

**Table 1:** Differential node analysis based on the degree centrality.

				**Degree centrality**			
	**θ Statistic**	***p*-value**	***q*-value**	**AST**	**OAST**	**ODG**	**GBM**
MAPK3	25.151	0.001	0.017	25	28.1	18.7	9.3
MAPK10	19.904	0.001	0.017	29	30.7	22.2	17.5
MAPK9	18.653	0.001	0.017	27.9	30.9	22.4	17.8
TOLLIP	17.877	0.002	0.026	25	28.2	20	15.3
TAB1	17.393	0.001	0.017	27.2	30.8	25.2	16.1
PIK3R1	17.098	0.001	0.017	28.9	30.7	24.5	18
AKT3	17.013	0.001	0.017	31.1	31.4	24.2	21.3
PIK3CB	15.215	0.002	0.026	29.1	31.8	23.9	21.7

*Statistical tests results of the comparison of four glioma subtypes on Toll like receptor signaling pathway geneset networks. Eight genes were selected as differentially coexpressed, by considering a q-value threshold at 0.05. AST, astrocytoma; OAST, oligoastrocytoma; ODG, oligodendroglioma; GBM, glioblastoma. BioNetStat identified genes on important cell regulatory pathways involved in gliomas formation*.

BioNetStat also includes a visual inspection of alterations in the correlation networks (heatmaps of the adjacency matrices). It also includes a list of the differences in the pairwise correlations, a table of variable set properties (e.g., spectral entropy, average node centrality, and average clustering coefficient) for each biological state, a rank of the centrality and local clustering coefficients, and a comparison of the measurements obtained in each state by heatmaps and boxplots. Also, BioNetStat provides a metabolic KEGG pathway view, using pathview R package. This functionality allows the user to visualize the gene expression, the concentration of proteins and metabolites, and the centrality of nodes at the KEGG pathway maps.

**Figure 1:**
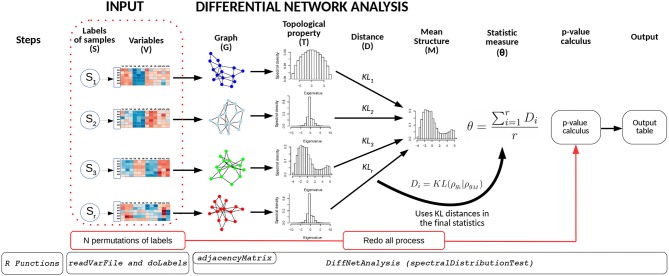
Schematic diagram of BioNetStat. BioNetStat receives an input file containing the values of the variables to be analyzed and *r* biological states (*S*_1_, …, *S*_*r*_). This figure illustrates the method performed with PDFG, however it can be replaced by centralities (such as Degree, Betweenness, and Closeness) without loss of generality.

The BioNetStat pipeline is summarized in Figure 1. For a detailed tutorial and manual, we refer the user to the Bioconductor page: doi: 10.18129/B9.bioc.BioNetStat.

### 2.7. Example Datasets

To illustrate the utility of BioNetStat, we considered two different datasets: (i) gene expression dataset from glioma, and (ii) a plant metabolism dataset. The first dataset was selected because the cancer gene expression data contain thousands of variables and hundreds of samples (common features in this area), allowing robust analysis. The second dataset was motivated by a large number of experiments in plant studies that use a small number of replicates.

The glioma dataset was obtained from a public database (TCGA) (Tomczak et al., 2015). The glioma is a brain tumor that occurs in glial cells, a tissue in charge of protecting and nourishing the neurons (Purves et al., 2001). We used gene expression data of 19,947 genes obtained from 612 samples divided into four cancer cell types: 174 oligodendroglioma samples, 169 astrocytoma samples, 114 oligoastrocytoma samples, and 155 glioblastoma multiforme (GBM) samples. The tumor tissues have different degrees of aggressiveness. GBM is the most aggressive, while astrocytoma, oligodendrogliomas, and oligoastrocytoma are less aggressive than GBM (Louis et al., 2016). To approximate the genes expression levels distribution to a normal distribution, we transformed the values by their logarithm to the base two. For constructing the correlation networks, we performed Spearman's independence test between each pair of genes and inserted an edge for those whose *p*-value is smaller than 0.05. The absolute Spearman's correlation coefficient weights all edges.

The plant metabolism dataset contains 73 metabolites from whole-plant sorghum development (de Souza et al., 2015). The data were obtained from five organs (leaves, culm, roots, prop roots, and grains) of six biological samples. We consider correlation graphs in which the edges are weighted by Pearson's correlations >0.75, as used by Jeong et al. (2001) and Ding et al. (2015).

## 3. Results and Discussion

To evaluate the performance of BioNetStat, we applied it on two datasets, namely glioma, and sorghum, and compared it to GSCA. The results for these comparisons are described in the following sections.

### 3.1. Analyses Using Glioma's Data Set

We performed Monte Carlo experiments to verify the ability of BioNetStat (based on the PDFG and the degree centrality) and GSCA to control the rate of false positives (control the proportion of type I error). We combined all 612 biological samples from four cancerous tissues (astrocytoma, oligoastrocytoma, oligodendroglioma, and GBM). For each test, we randomly selected, from a uniform distribution, 120 biological samples, and 50 genes to build each network. Thus, we consider that they come from the same dataset (i.e., under the null hypothesis). To analyze the results, we estimated the proportion of false positives to each *p*-value threshold. We analyzed the performance of the three methods (BioNetStat based on the PDFG and the degree centrality, and GSCA) when comparing five and ten networks (Figures S1A,B). Under the null hypothesis, we expect that the observed proportion of false positives is similar to the expected proportion set by the *p*-value threshold. In Figure S1, we observe that all methods indeed control the rate of false positives as expected.

To measure the statistical power (the ability to detect differences among two or more networks when indeed they are different) of the methods, we build *r* networks similarly to described in the previous paragraph. However, for one of the networks, we permuted the measurements of some gene expressions to change its co-expression pattern. The proportion of permuted genes is denoted by γ. In other words, for one of the networks we set γ > 0 (the network is different from the others) and γ = 0 for the others. Therefore, we expect that the methods detect that there is a different network. Then, to estimate the rate of false positives, we apply the tests in two networks selected from the *r*−1 networks that are under the null hypothesis (γ = 0). Here, we expect to obtain a rate of false positives similar to the level of significance set by the *p*-value threshold. We carried out this experiment 1,000 times for different proportions of altered genes (γ = 0.05, 0.1, 0.2, 0.3, 0.5) and number of networks (*r* = 2, 3, 5, 10, 15, 20).

To summarize the statistical power of the test, we constructed Receiver Operating Characteristic (ROC) curves. The *x* and *y* axes of the ROC curves are the empirical false and true positive rates, respectively. The area under this ROC curve (AUC) summarizes the empirical power of the test. Under the alternative hypothesis (when at least one of the networks are generated by a different model), we expect that the proposed test present a ROC curve above the diagonal and consequently an AUC > 0.5.

**Figure 2:**
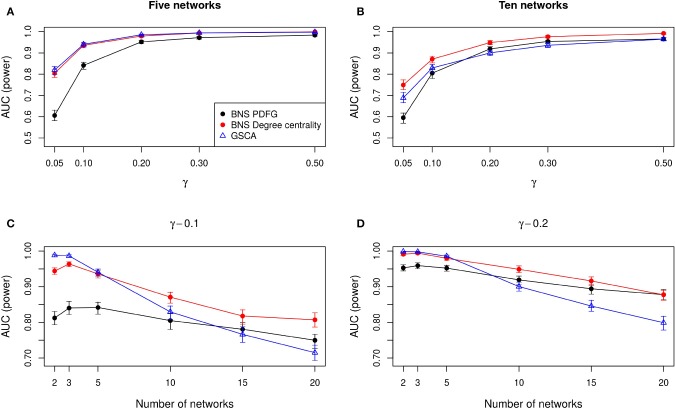
Comparison of the statistical power of BioNetStat based on PDFG (black circles) and degree centrality (red circles), and GSCA (blue triangles). The values in the *y*-axis represent the areas under the ROC curves, considering the confidence interval of 95%. In **(A,B)**, the *x*-axis represents the ratio of perturbed genes (γ), for the comparison of 5 and 10 networks, respectively. In **(C,D)**, the *x*-axis represents the number of compared networks, which varies from two to 20, by fixing the γ to 0.1 and 0.2, respectively. Observe in **(A,B)** that both BioNetStat and GSCA statistical power increases proportionally to the increase of γ. We also observed that for a fixed γ, the empirical power decreases with the increase of the number of networks, as shown in **(C,D)**. Furthermore, we observe that the empirical power of GSCA decreases faster than BioNetStat with the increase of the number of networks.

In Figures 2A,B, we show the AUC when we compare five and ten biological states/networks (denoted by *r*), respectively, to γ = 0.05, 0.1, 0.2, 0.3, 0.5. In Figures 2C,D, we show the AUC for each *r* = 2, 3, 5, 10, 15, 20, and a fixed γ = 0.1, 0.2, respectively.

As expected, we observe in Figures 2A,B that both BioNetStat (based on PDFG and the degree centrality) and GSCA increase the statistical power proportionally to the increase of γ. Moreover, the performance of BioNetStat based on the PDFG presented lower power than BioNetStat based on the degree centrality and GSCA for 0.05 ≤ γ ≤ 0.2 (Figure 2A). By comparing ten networks, we observe that the power of GSCA becomes lower than BioNetStat based on the degree centrality for γ ≥ 0.05, and similar to BioNetStat based on PDFG for γ ≥ 0.2 (Figure 2B).

We also observed that for a fixed γ, the empirical power decreases with the increase of the number of networks, as shown in Figures 2C,D. By comparing the performance of the methods, we observe that the empirical power of GSCA is greater than BioNetStat when the number of networks is small (*r* = 2, 3) and the changes in the networks are moderate (γ = 0.1) (Figure 2C). When the number of networks is five, the performance of BioNetStat based on the degree centrality is similar to GSCA for the two evaluated values of γ (Figures 2C,D). When the number of networks is >10 and γ ≥ 0.2, the power of BioNetStat based on PDFG becomes greater than GSCA. Furthermore, we observe that the empirical power of GSCA decreases faster than BioNetStat with the increase of the number of networks.

Besides the statistical power, other criteria are relevant in the choice of the method to be used. In the following steps, we further analyze the glioma dataset.

We applied BioNetStat based on PDFG and GSCA in the glioma dataset comparing gene co-expression networks across the glioma types. We defined gene sets according to the canonical pathways obtained from Molecular signature Database Collection v5 (Subramanian et al., 2005). That database contains 1,329 canonical pathways. We performed the tests only with the subsets that presented at least 10 genes. Then, we tested the 1,289 gene sets.

We show the results of the tests, each one based on 1,000 permutation tests, for all gene sets in Supplementary Data Sheet 1. For the significance values (α) equal to 0.05 and 0.1, the total number of gene sets, which has at least one network statistically different from each other, were 490 and 801, respectively. One hundred and twenty-two, and 305 gene sets were co-identified by both methods considering a *q*-value of 0.05 and 0.1, respectively. For α = 0.05 and α = 0.1, BioNetStat identified, respectively, 62 and 79 gene sets that were not identified by GSCA. The latter identified, respectively, 306 and 417 gene sets that were not identified by BioNetStat. Thus, these results suggest that BioNetStat obtains results complementary to GSCA.

This complementarity is already expected, because GSCA and BioNetStat present different statistical tests. GSCA compares the Euclidean distances among matrices. It performs the pairwise comparison, edge by edge, being more sensitive to localized changes (few edges modifications) in networks, while BioNetStat is more adequate for differences spread across the correlation matrix. On the other hand, methods such as CoGA (Santos et al., 2015) and GSCNA (Rahmatallah et al., 2014) compare networks based on their overall structures, such as eigenvector centrality and spectral distributions. These strategies do not detect local changes in the network, since structural properties may remain unaffected. Rahmatallah et al. (2014) stated that GSCNA detects alterations when the major players such as genes of signaling pathways change across the different biological states, whereas GSCA detects these modifications when the average correlation changes (Rahmatallah et al., 2014), such as in pathways related to metabolism. As BioNetStat is based on topological features of the network, we expect that it would detect changes in signaling pathways rather than pathways related to metabolism.

To verify this hypothesis, we classified the 1,289 gene sets in *signaling* or *non signaling* pathways and compared the performance of GSCA against BioNetStat. To classify as *signaling* pathway, we searched for key terms in gene sets such as “signal,” “cascade,” “receptor,” “activ*,” “regula*,” “pid,” “ach,” “arrestin,” and the transcription factor names obtained from MsigDB website. The proportion of signaling pathways in the 1,289 gene sets is 51.2%. Only the gene sets selected by each method for a *q*-value threshold at 0.05 were considered. Our test classified 52.8% of the selected gene sets by GSCA as signaling pathways. Whereas, for BioNetStat, the test selected 59.2% out of 184 gene sets as signaling pathways. We performed the proportion method (prop.testR function), considering the null hypothesis that measured proportion is equal to 51.2% and the alternative that the measured proportion is greater than 51.2%. Only BioNetStat presented a proportion of signaling pathways statistically greater than the entire dataset (*p* = 0.018), whereas GSCA did not (*p* = 0.269). Therefore, as expected, BioNetStat detects more changes in signaling pathways than GSCA.

To highlight the applicability of the proposed method, we went deeper in the analysis of the 62 gene sets that were detected by BioNetStat, but not by GSCA, considering a *q*-value threshold at 0.05. Among this 62 differentially coexpressed gene sets, 38 were classified as signaling pathways. We searched for a gene set that contained NFκB gene, a transcription factor which controls more than a hundred of genes, well-known to be associated with glioma's formation (Mieczkowski et al., 2015; Kinker et al., 2016; Ferrandez et al., 2018). Then, we selected “KEGG TOLL-LIKE RECEPTOR SIGNALING PATHWAY.” Also, Toll-like receptors (TLRs) is an important gene set, part of a signaling pathway gene set associated with gliomas (Ferrandez et al., 2018). TLRs are membrane-bound receptors, which serve as crucial pattern recognition receptors with central roles in the induction of innate immune responses (Kawai and Akira, 2007). Pathogen recognition by TLRs provokes rapid activation of innate immunity by inducing production of proinflammatory cytokines and upregulation of costimulatory molecules (Ferrandez et al., 2018). Therefore, the TLR genes trigger a signaling chain reaction that leads to NFκB activation which, in turn, triggers inflammatory responses (Kawai and Akira, 2007).

**Figure 3:**
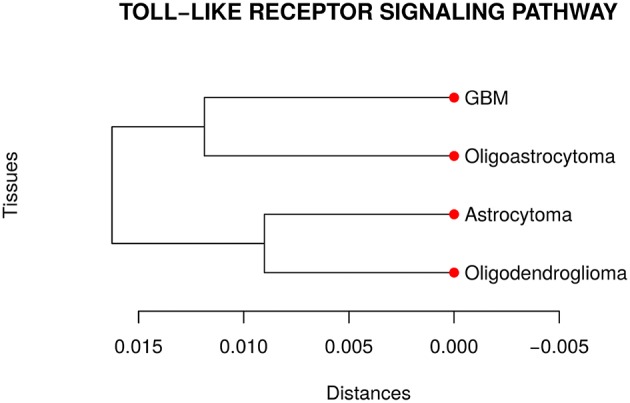
Dendrogram of the distances among the four glioma subtypes regarding the *Toll like receptor signaling pathway*. The unexpected closeness between Oligoatrocytoma and GBM is probably due to the specific features in tissues characterizations.

Our analyses suggested that at least one network is different from the others in the TLR gene set. Then, we performed a pairwise comparison of the four cancer types to understand better how they differ from each other. Figure 3 presents the dendrogram obtained by calculating the pairwise Jensen-Shannon divergence (a symmetric version of KL divergence to pairwise comparison) between the networks. We expected that the most aggressive cancer type, namely GBM, be in one branch and the other three types, on another branch. However, the cancer types GBM and oligoastrocytoma are in one branch and oligodendroglioma, and astrocytoma are in another branch. The unexpected closeness between GBM and oligoastrocytoma could be a consequence of a confusing clinic classification method of gliomas subtypes. The TCGA database classifies gliomas only into four types *astrocytoma, oligoastrocytoma, oligodendroglioma*, and GBM. However, there is a more aggressive type of *oligoastrocytoma*, called *anaplastic oligoastrocytoma*, that can also be classified as a glioblastoma with an oligodendroglial component (Nakamura et al., 2011). Since 2007, the World Health Organization (WHO) defines the *anaplastic oligoastrocytoma* as a Glioblastoma (Marucci, 2011). Therefore, there must be intermediate states between both types (Oligoastrocytoma and GBM), not discriminated in our data, that explain this closeness between them.

BioNetStat also allows us to identify in which node the connections change significantly by the *differential node analysis*. We performed this analysis by using the degree centrality. The *TLR signaling pathway* presented statistically significant changes of nodes degree centrality (θ = 2.88 and *p* = 0.027). In this gene set, eight genes presented their degree centrality significantly altered (Table 1). Three of them are mitogen-activated protein kinase MAPK (3, 9, and 10) and are integrated into the RAS/MAPK signaling pathway. When RAS (Rat Sarcoma) genes are active, they regulate the MAPK pathway and vital processes into the cell, such as proliferation, differentiation, signal transduction, apoptosis, and tumorigenesis (Mao et al., 2013). Modifications in this pathway could lead to abnormal function of these processes. As an example, the overexpression of RAS was detected in astrocytoma and GBM (Mao et al., 2013). Other three genes differentially coexpressed are in the PIK3-PTEN-Akt-mTOR pathway. The genes PIK3 indirectly activates Akt which, in turn, activates mTOR (mammalian target of rapamycin). This gene cascade leads to an integration of upstream signals into effector actions, controlling multiple downstream targets involved in cell growth and division. Most of the genes differently coexpressed such as MAPKs, PIK3s, and AKT3 are involved into the gliomas formation (the PIK3 pathway is altered in about 70% of GBMs) (Mao et al., 2013), demonstrating the importance of gene set detected by BioNetStat.

### 3.2. Analyses Using *Sorghum bicolor*'s Data Set

In the second data set, we studied how the metabolic networks of five plant organs differ from each other. The 73 metabolites analyzed in sorghum organs (leaf, culm, root, prop root, and grains) were partitioned in five groups according to their biochemical roles: carbohydrates, amino acids, organic acids, nucleotides, and all 73 metabolites. We built one network for each organ and each metabolic group. Then we compared the networks across the organs using the PDFG, the centrality tests of BioNetStat, and GSCA method.

The grain-filling stage in plants is largely dependent on metabolic status (Schnyder, 1993). Thus, it is important to understand to what extent the metabolic networks in distinct organs differ from each other. de Souza et al. (2015) investigated whether each organ performs a specific role in plant metabolism during the grain-filling in sorghum plants. Here, we complemented their study by analyzing the same dataset based on a systemic point of view and network modeling. First, we investigated if the PDFG and degree centralities are different among the networks (organs). Table 2 presents the results of PDFG, degree centrality, and GSCA tests. Comparing the metabolic networks structures, through their PDFG, it can be observed that at least one organ is different from the others, regarding the *all metabolites* and the *carbohydrates* set. According to the degree centrality analysis, the organs networks are significantly different in the five metabolites sets. GSCA detected the *organic acids* and the *nucleotides* sets as differentially coexpressed. Analyzing the concentrations of metabolites, de Souza et al. (2015) also found differences among organs in the four metabolites sets.

**Table 2:** Results of the PDFG and degree centrality statistical tests comparing all five organs networks.

		**PDFG**			**Degree centrality**			**GSCA**		
**Name**	**Size**	**θ Statistic**	***p*-value**	***q*-value**	**θ Statistic**	***p*-value**	***q*-value**	**θ Statistic**	***p*-value**	***q*-value**
All	73	0.017	**0.006**	**0.015**	17.167	**0.001**	**0.002**	0.329	**0.006**	**0.042**
Carbohydrate	18	0.056	**0.003**	**0.015**	3.857	**0.001**	**0.002**	0.299	0.416	0.416
Organic acid	13	0.044	0.065	0.108	3.482	**0.001**	**0.002**	0.341	**0.019**	**0.044**
Amino acid	24	0.018	0.292	0.312	5.152	**0.003**	**0.004**	0.314	0.179	0.313
Nucleotide	12	0.034	0.312	0.312	3.041	**0.006**	**0.006**	0.352	**0.019**	**0.044**

*The q-values <0.05 are in bold*.

**Figure 4:**
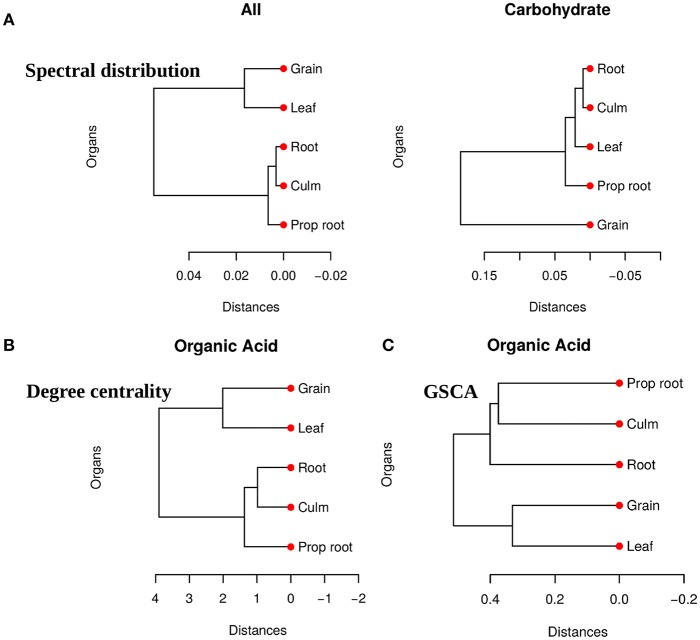
**(A)** Dendrogram of distances among five network PDFG for the groups of *all metabolites* and *carbohydrates*. **(B)** Dendrogram of distances among five network degree cetrality of the organs for the *organic acid* metabolites. **(C)** Dendrogram of distances among five network of the organs for the *organic acid* metabolites measured by GSCA. We can conclude that the grain network is more distant network from the others, reinforcing that this organ has a different metabolism.

We obtained pairwise distances among the organ networks for those metabolic sets with a statistically significant difference. Figure 4A shows the distances among networks according to Jensen-Shannon divergence. Considering the *all metabolites* network, the grain is significantly different from the culm, prop root, and roots (Table S1). Additionally, according to the *carbohydrate* results, the metabolism of the grains is different from all other organs (Table S2). The results suggest that the grain has a specific metabolic structure and that the leaf network is more similar to the grain network than to the other organs. Considering that the grain is the main sink of the plant during the grain-filling (period of the experimental harvest of the studied data) (de Souza et al., 2015), we expected that its metabolism to be different from other organs.

For the tests performed with the degree centrality, we identified significant differences in all groups. The results suggest that even if the network structure (PDFG) does not change, the role of the metabolites and its mean correlation values in each organ can be different. The *organic acid*, identified by BioNetStat degree centrality network and GSCA can exemplify this phenomenon. According to both methods, the grain and leaf networks are the most distant (Figures 4B,C) and statistically different from the remaining organs (Tables S3, S4). For this reason, we investigated which nodes changed the degree centrality value in the *organic acids* network among the organs (Table 3), by performing the *differential node analysis*. The GSCA has not implemented a similar method capable of comparing whether the importance of the nodes changes among states. Therefore, we forwarded the analysis using only BioNetStat.

**Table 3:** Differential node analysis based on degree centrality.

				**Degree centrality**				
**Metabolite**	**θ Statistic**	***p*-value**	***q*-value**	**Leaf**	**Culm**	**Root**	**Prop root**	**Grain**
Piruvate	4.219	0.001	0.003	10.328	1.906	0.765	0.821	9.579
Mevalonate	4.215	0.001	0.003	9.93	0	0.838	0	8.191
cis-Aconitate	3.582	0.001	0.003	9.361	0	0.872	0.821	6.693
AKG	3.499	0.001	0.003	9.474	2.641	0.872	5.11	10.854
2/3PGA	3.862	0.001	0.003	10.412	0.805	5.216	0	9.695
Chiquimate	3.523	0.002	0.004	8.97	0	0.913	2.517	7.994
Malate	3.029	0.003	0.005	10.374	1.917	0.765	5.206	7.376
Isocitrate	2.782	0.003	0.005	9.588	1.872	4.654	5.316	9.898
Citrate	2.631	0.009	0.013	10.019	1.857	2.702	6.104	7.156
PEP	2.205	0.064	0.083	9.393	1.863	3.583	5.111	2.529
Fumarate	2.387	0.089	0.105	4.018	1.845	3.505	0.829	10.01
trans-Aconitate	2.712	0.097	0.105	0	1.842	0.913	3.304	9.835
Succinate	2.115	0.141	0.141	8.871	1.84	4.567	6.22	7.738

*Statistical tests results of comparison among five organs on the 13 organic acids. The table shows the metabolite's name, the θ statistic, the nominal p-value, the adjusted p-value for tests performed (q-value), and the degree centrality of each node in the five organs*.

**Figure 5:**
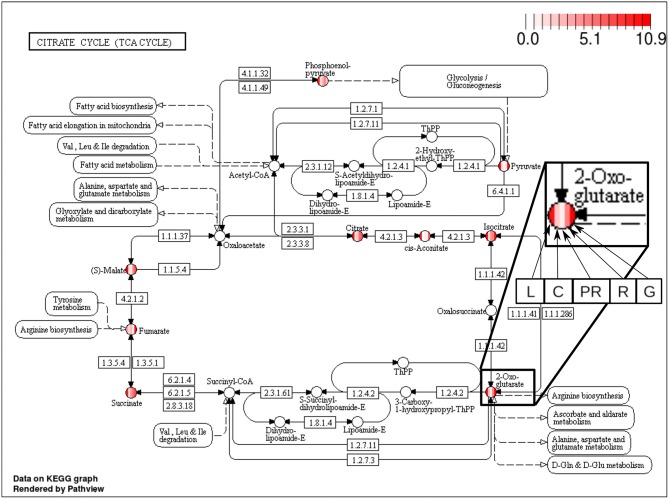
Citrate cycle (TCA Cycle) metabolic pathway from KEGG database (Kanehisa and Goto, 2000). Each metabolite, represented by the circles, is partitioned in five columns, in which each one represents one of the organs, leaf (L), culm (C), prop root (PR), root (R), and grains (G), from the left to right, as shown in the highlighted node of 2-Oxoglutarate (AKG) at the right of the figure. The color of the columns represents the degree centrality value of that metabolite in the organ network. The values of degree centrality in this figure vary from 0 to 10.9.

The majority of the metabolites of the *organic acids* dataset belong to the citrate cycle (or Krebs cycle), a chain of reactions that transfer energy (by electrons transferring) from complete pyruvate oxidation to cofactors used in ATP production (Siedow and Day, 2000). The network of the *organic acid* is more connected in the leaf and grain than in the culm, prop root and root. The average degree centrality in the leaf and grain is 8.51 and 8.27, respectively, whereas in the culm, prop root, and root networks the average degree centrality is 1.41, 3.18, and 2.32, respectively (extracted from Table 3). The metabolites with highest degree centrality in the leaf and in the grain are the pyruvate and the AKG (α-ketoglutarate), respectively (Table 3). These results are in agreement with previous observations by de Souza et al. (2015) that pointed out pyruvate as a central molecule in metabolism, connecting the citrate cycle with many other pathways. Our network analysis using BioNetStat revealed that the AKG is also a relevant metabolite, being a precursor of many amino acids synthesis pathways (Figure 5) (Siedow and Day, 2000).

The analyzed data were collected between 10 a.m. and 12 a.m. when the leaf performs constant photosynthesis and mobilization of carbon. Also, the grain metabolism is geared toward storage of carbohydrate and proteins. Therefore, we have evidence to believe that the average degree centrality of metabolites are higher in the leaf and grain networks because the organic acid metabolism of these organs is more active than the organic acid metabolism of the other organs. Our findings reinforce that network analysis brings a new view to the data, since de Souza et al. (2015) did not find these molecules in comparisons among organ metabolisms, as highly concentrated in these organs. Furthermore, to highlight relevant variables in the system, BioNetStat performs the *differential node analysis*, a method not available in other tools considered in this work.

## 4. Conclusion

BioNetStat is a network analysis Bioconductor package, containing a Graphical User Interface, that allows the comparison of two or more correlations networks. The proposed method is an adaptation and generalization of CoGA, which aims to meet demand on multistate experiments. We show here that BioNetStat performs the *differential network analysis*, exploring networks features and highlighting the main differences among states. Moreover, it carries out statistical tests to estimate the significance of the results. We showed that all the statistical tests performed by BioNetStat effectively control the rate of false positives. Our simulation experiments and applications in real datasets suggest that BioNetStat complements and advances previous tools (CoGA and GSNCA) for differential co-expression analysis, i.e., BioNetStat allows the comparison of more than two networks simultaneously. We also conclude that BioNetStat is less sensitive to the increase in the number of networks than GSCA. Furthermore, it is able to identify more gene sets associated with important signaling pathways than GSCA, and also highlights key genes in the networks (centrality analyses). The study cases show that BioNetStat helps to find differences beyond the analysis of the network, highlighting features that can be biologically supported while undetected by in orthodox analyses. BioNetStat provides numerical results combined with visual inspection in the graphical user interface that might be helpful in the identification of critical elements of the analyzed system. BioNetStat is not restricted to analyses of genes coexpression networks. Differently from other tools, BioNetStat can be used with different types of data sets such as the ones generated by metabolomics, proteomics, phenomics, and possibly social and economic data.

## Data Availability

Publicly available datasets were analyzed in this study. This data can be found here: https://portal.gdc.cancer.gov.

## Author Contributions

VJ, SS, AF, and MB conceived and designed the experiments, analyzed the data, and wrote the paper. VJ performed the experiments.

### Conflict of Interest Statement

The authors declare that the research was conducted in the absence of any commercial or financial relationships that could be construed as a potential conflict of interest. The handling editor declared a shared affiliation, though no other collaboration, with the authors at the time of the review.
