# Supplementary material for: BioNetStat: A Tool for Biological Networks Differential Analysis
Source: Front Genet. 2019 Jun 21;10:594. doi: 10.3389/fgene.2019.00594 (PMC6598498; doi:10.3389/fgene.2019.00594)
Supplement: Supplementary file 2 [file Data_Sheet_2.pdf]

Supplementary Material: BioNetStat: A tool for biological networks differential analysis

1 TABLES OF CENTRALITIES

Let $G = (V, E)$ be an undirected graph with n_V nodes and n_E edges, and $\mathbf{A}$ be the adjacency matrix of G. The distance (number of edges in the shortest path) between two nodes v_i and v_j is denoted $d(v_i, d_j)$. For each node or edge i, define $p_i(v_j, v_k)$ as 1 if the shortest path between nodes v_j and v_k pass trough i, and zero otherwise.

We define measures of structural properties of graph G in Table S1.

Table S1. Summary of node/edge centrality measures. The measures include degree centrality, eigenvector centrality, closeness centrality, betweenness centrality, clustering coefficient and edge betweenness.

Importance measure	Definition	Mathematical definition
Degree Centrality (Barabási and Oltvai, 2004)	Number of edges connected to a node.	$C_G(v_i) = \sum_{j=1, j\neq i}^{n_v} \mathbf{A}_{ij}$
Eigenvector Centrality (Bonacich, 1972)	The eigenvector centrality of a node is proportional to the centralities of its neighbors, weighted by the strength of the connections	$x_i = \frac{1}{\lambda} \sum_{j=1}^{n_v} \mathbf{A}_{ij} x_j$ $\mathbf{A}\mathbf{x} = \lambda \mathbf{x}$
Closeness Centrality (Freeman, 1978)	It measures the average proximity of a node from all others nodes of the network.	$C_c(v_i) = 1/ \sum_{j=1, j\neq i}^{n_v} d(v_i, d_j)$
Betweenness Centrality (Freeman, 1978)	It measures the importance of a node in the network communication, by counting how many shortest paths pass through the node.	$C_b(v_i) = \sum_{j=1, j\neq i}^{n_v} \sum_{k=j+1, k\neq i}^{n_v} p_i(v_j, v_k)$
Clustering coeficient (Watts and Strogatz, 1998)	It quantifies how connected the neighbors of a certain node are.	$Cc(v_i) = \frac{\sum_{v_j, v_k \epsilon N_i} \mathbf{A}_{jk}}{k_i(k_i - 1)}$
Edge betweenness (Girvan and Newman, 2002)	It measures the importance of an edge in the network communication, by counting how many shortest paths pass through the edge.	$E_b(e_i) = \sum_{j=1, j\neq i}^{n_v} \sum_{k=j+1, k\neq i}^{n_v} p_i(v_j, v_k)$

2 CONTROL OF FALSE POSITIVE RATE

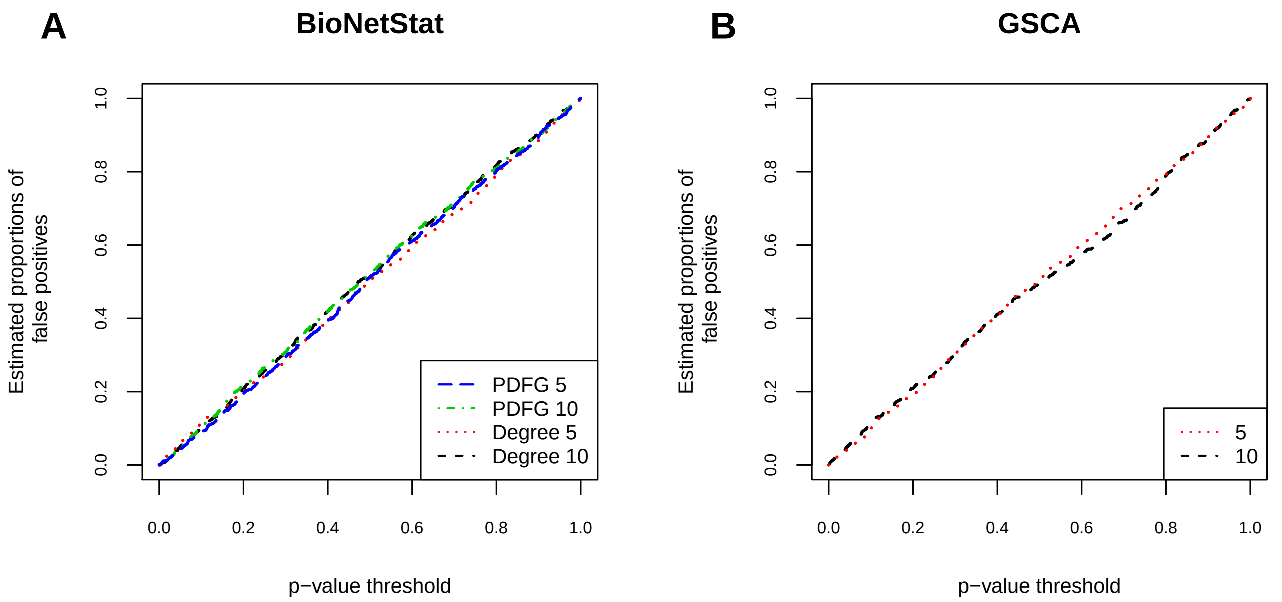

Figure S1. False positives rate for BioNetStat (A) based on the PDFG and degree centrality, and for GSCA (B). In panels A and B, the x-axis represents the p-value threshold and the y-axis represents the estimated proportion of false positives. If both proportions are similar, we can state that the method indeed controls the false positive rate. Moreover, we also show that this control is effective, independent of the number of networks (five and ten networks).

3 TABLES OF SORGHUM ORGANS PAIRWISE COMPARISON

Table S2. Pairwise distances among five network spectral distributions of the organs for the groups of *all metabolites*. In the upper-right triangle are the Jensen Shannon divergence value related to comparisons between organs. In the left-down triangle are the pvalue resulting of comparison test. The pvalues less than 0.05 are in bold.

**All**	Leaf	Culm	Prop root	Root	Grain
Leaf	-	0.023	0.03	0.022	0.017
Culm	0.115	-	0.006	0.003	0.048
Prop root	0.055	0.973	-	0.007	0.055
Root	0.15	0.997	0.965	-	0.046
Grain	0.34	**0.017**	**0.005**	**0.01**	-

Table S3. Pairwise distances among five network spectral distributions of the organs for the groups of *carbohydrates*. In the upper-right triangle are the Jensen Shannon divergence value related to comparisons between organs. In the left-down triangle are the pvalue resulting of comparison test. The pvalues less than 0.05 are in bold.

**Carbohydrate**	Leaf	Culm	Prop root	Root	Grain
Leaf	-	0.021	0.035	0.02	0.17
Culm	0.516	-	0.019	0.01	0.172
Prop root	0.17	0.784	-	0.034	0.168
Root	0.601	0.997	0.599	-	0.184
Grain	**0.015**	**0.01**	**0.005**	**0.01**	-

Table S4. Pairwise distances among five network degree centralities of the organs for the groups of *organic acids*. In the upper-right triangle are the euclidean distance value related to comparisons between organs. In the left-down triangle are the pvalue resulting of comparison test. The pvalues less than 0.05 are in bold.

**Organic Acids**	Leaf	Culm	Prop root	Root	Grain
Leaf	-	3.894	3.216	3.496	2.021
Culm	**0.005**	-	1.305	0.98	3.587
Prop root	**0.01**	0.433	-	1.374	3.095
Root	**0.007**	0.707	0.409	-	3.28
Grain	0.117	**0.007**	**0.007**	**0.012**	-

Table S5. Pairwise distances among five network calculated by GSCA of the organs for the groups of *organic acids*. In the upper-right triangle are the euclidean distance value related to comparisons between organs. In the left-down triangle are the pvalue resulting of comparison test. The pvalues less than 0.05 are in bold.

**Organic Acids**	Leaf	Culm	Prop root	Root	Grain
Leaf	-	0.513	0.483	0.457	0.332
Culm	**0.001**	-	0.375	0.391	0.478
Prop root	**0.004**	**0.015**	-	0.401	0.438
Root	0.051	**0.001**	0.092	-	0.425
Grain	0.073	**0.001**	**0.001**	**0.001**	-

REFERENCES

Barabási, A.-L. and Oltvai, Z. N. (2004). Network biology: understanding the cell's functional organization. *Nature reviews. Genetics* 5, 101–113. doi:10.1038/nrg1272

Bonacich, P. (1972). Bonacich_1972_Technique for Analyzing Overlapping Memberships. *Sociological Methodology* 4, 176–185

Freeman, L. C. (1978). Centrality in social networks conceptual clarification. *Social Networks* 1, 215–239. doi:10.1016/0378-8733(78)90021-7

Girvan, M. and Newman, M. E. J. (2002). Community structure in social and biological networks. *Proceedings of the National Academy of Sciences of the United States of America* 99, 7821–7826. doi:10.1073/pnas.122653799

Watts, D. J. and Strogatz, S. H. (1998). Collective dynamics of'small-world' networks. *Nature* 393, 440–442. doi:Doi10.1038/30918
